# A truncated peptide Spgillcin_177–189_ derived from mud crab *Scylla paramamosain* exerting multiple antibacterial activities

**DOI:** 10.3389/fcimb.2022.928220

**Published:** 2022-08-02

**Authors:** Xiaofei Wang, Xiao Hong, Fangyi Chen, Ke-Jian Wang

**Affiliations:** ^1^ State Key Laboratory of Marine Environmental Science, College of Ocean and Earth Sciences, Xiamen University, Xiamen, China; ^2^ State-Province Joint Engineering Laboratory of Marine Bioproducts and Technology, College of Ocean and Earth Sciences, College of Ocean and Earth Sciences, Xiamen University, Xiamen, China; ^3^ Fujian Innovation Research Institute for Marine Biological Antimicrobial Peptide Industrial Technology, College of Ocean and Earth Sciences, Xiamen University, Xiamen, China

**Keywords:** *Scylla paramamosain*, antimicrobial peptide, Spgillcin_177–189_, drug resistance, *S. aureus* and *P. aeruginosa*

## Abstract

Antimicrobial peptides (AMPs) may be the most promising substitute for antibiotics due to their effective bactericidal activity and multiple antimicrobial modes against pathogenic bacteria. In this study, a new functional gene named *Spgillcin* was identified in *Scylla paramamosain*, which encoded 216 amino acids of mature peptide. *In vivo*, Spgillcin was dominantly expressed in the gills of male and female crabs, offering the highest expression level among all tested organs or tissues. The expression pattern of Spgillcin was significantly altered when challenged by *Staphylococcus aureus*, indicating a positive immune response. *In vitro*, a functional truncated peptide Spgillcin_177–189_ derived from the amino acid sequence of Spgillcin was synthesized and showed a broad-spectrum and potent antibacterial activity against several bacterial strains, including the clinical isolates of multidrug-resistant (MDR) strains, with a range of minimum inhibitory concentrations from 1.5 to 48 μM. Spgillcin_177–189_ also showed rapid bactericidal kinetics for *S. aureus* and *Pseudomonas aeruginosa* but did not display any cytotoxicity to mammalian cells and maintained its antimicrobial activity in different conditions. Mechanistic studies indicated that Spgillcin_177–189_ was mainly involved in the disruption of cell membrane integrity where the membrane components lipoteichoic acid and lipopolysaccharide could significantly inhibit the antimicrobial activity in a dose-dependent manner. In addition, Spgillcin_177–189_ could change the membrane permeability and cause the accumulation of intracellular reactive oxygen species. No resistance was generated to Spgillcin_177–189_ when the clinical isolates of methicillin-resistant *S. aureus* and MDR *P. aeruginosa* were treated with Spgillcin_177–189_ and then subjected to a long term of continuous culturing for 50 days. In addition, Spgillcin_177–189_ exerted a strong anti-biofilm activity by inhibiting biofilm formation and was also effective at killing extracellular *S. aureus* in the cultural supernatant of RAW 264.7 cells. Taken together, Spgillcin_177–189_ has strong potential as a substitute for antibiotics in future aquaculture and medical applications.

## Introduction

There is no doubt that antibiotics have been widely used in the world since their discovery (Browne et al., 2020). Unfortunately, the extensive use and frequent abuse of antibiotics lead to the increasing prominence of antibiotic resistance (O’Neill, 2016). In particular, antibiotic resistance is a new emerging serious threat to public health as seen in aggravated clinical diseases such as pneumonia (Chalmers et al., 2014), urinary tract infections (Kahlmeter and Eco.Sens, 2003), and other common ailments. The need for new antibacterial substances that can be substituted for antibiotics has received universal scientific recognition.

Antimicrobial peptides (AMPs) are short peptides that exhibit antimicrobial and other immune properties (Brogden, 2005; Seo et al., 2012; Magana et al., 2020). As an important component of innate immunity, AMPs feature diverse sources and an extensive distribution. To date (25 April 2022), 3,324 AMPs have been listed in the Antimicrobial Peptide Database (http://aps.unmc.edu/). The antimicrobial mechanisms of AMPs usually manifest as membrane disruption through electrostatic interactions, altered permeability of cell membranes, and leakage of cell contents. In some cases, AMPs target intracellular substances and interfere in key cellular processes, such as binding DNA and RNA and interacting with proteins (Brogden, 2005; Ho et al., 2016). The different mechanisms of action conferred by AMPs enrich the prospects of their application compared to some traditional antibiotics that usually focus on a single target, such as a single enzyme for which drug’s potency is easily decreased (Blair et al., 2015; Bai et al., 2021). The emergence of multidrug-resistant (MDR) pathogens creates a potential crisis in antibiotic use and raises fears of untreatable infections. However, bacteria that have acquired antibiotic resistance still display sensitivity to AMPs, suggesting that it is more difficult to induce resistance to them (de Breij et al., 2018; Bai et al., 2021). Moreover, it is known that the biofilms covering bacterial surfaces account for many chronic and recurrent infections on which antibiotics usually have less effects (Hoiby et al., 2010; Kranjec et al., 2021), and AMPs are reported to possess potent anti-biofilm activities (Casciaro et al., 2020; Zhang et al., 2022).

At present, to meet research and clinical applications, the discovery of novel AMPs in various organisms is a subject of continuous research (Toda et al., 2019; Rued et al., 2021). Of the known AMPs, very few (approximately 4% among 3,324 AMPs) have been identified in marine animals (Tincu and Taylor, 2004; Masso-Silva and Diamond, 2014), yet marine animals live in a very complex aquatic environment with abundant and varied microorganisms, harmful contaminants, and water eutrophication pressures, and thus the characterization of new AMPs that might be special or unique in marine animals is necessary. AMPs in marine animals may be different from those of land animals, and some AMPs, like Scygonadin, reportedly only exist in the mud crab *S. paramamosain* (Huang et al., 2006; Wang et al., 2007). *S. paramamosain* is often studied, and not only they are crustaceans that only rely on the innate immune system to defend themselves from foreign pathogen invasion but also they depend on the distinctive growth and development characteristics of mud crabs, which has over 20 episodes of molting from embryo to maturity. During each ecdysis, the crabs are more vulnerable and undoubtedly confront the serious threat of suffering infections. Considering these features of mud crabs, it is likely that more effective antibacterial components like AMPs may exist in the crabs during this developmental process. Since the first AMP in crabs was identified in 1996 (Schnapp et al., 1996), several dozens of AMPs have been successively reported (Stensvag et al., 2008; Sun et al., 2015). The well-known crustin family AMPs in crabs or shrimps has been extensively studied (Smith et al., 2008), and there are now more than 200 crustin peptide homologues documented in NCBI. Those published crustins have a broad antimicrobial spectrum and strong antimicrobial properties (Rekha et al., 2019; Wang et al., 2021). In recent years, a dozen AMPs have been identified in the reproductive tissues of mud crabs (Huang et al., 2006; Wang et al., 2007; Yang et al., 2020)—for instance, the Scygonadin family plays an important role in reproductive immunity (Xu et al., 2011a; Xu et al., 2011b). Interestingly, the antimicrobial activity of each reported AMP in crabs is distinctive, and some peptides like crustin (Rekha et al., 2018), identified in the crab *Portunus pelagicus*, show a moderate or weak antimicrobial activity, whereas others like Scyreprocin, recently identified in mud crabs, have potent activities against bacteria and fungi (Yang et al., 2020). The fact that each AMP has a distinctive limited antibacterial spectrum motivates researchers to continuously explore unknown AMPs from marine crabs or other crustaceans.

In our previous study investigating the modulated immune-associated genes in mud crabs challenged with *Vibrio alginolyticus* using transcriptome analysis, one functional gene encoding a 216-amino-acid protein was screened, and this gene presented a positive response against *V. alginolyticus* stimulation. Considering that this gene showed the highest expression level in the gills of male and female crabs, thus we named it “Spgillcin”. The truncated peptide Spgillcin_177–189_ derived from the sequence of Spgillcin was synthesized and exhibited potent antimicrobial activities against several strains, including *S. aureus*, *P. aeruginosa*, *A. baumannii*, and *E. coli*. Mechanistic studies showed that Spgillcin_177–189_ disrupted membrane integrity, changed membrane permeability, and ultimately caused cell death. Collectively, the truncated peptide Spgillcin_177–189_ was the structural domain derived from *Spgillcin* gene sequence and showed antimicrobial activity. Furthermore, bacterial resistance to Spgillcin_177–189_ was determined by culturing a serial passage of methicillin-resistant *S. aureus* (MRSA) and MDR *P. aeruginosa* for 50 days. Moreover, the antibiofilm activity of Spgillcin_177–189_ was evaluated by measuring *S. aureus* and *P. aeruginosa* biofilm mass. Finally, the killing effect on extracellular *S. aureus* in the cultural supernatant of RAW 264.7 cells was preliminarily assessed.

## Materials and methods

### Animals and bacterial strains

Mud crabs (*S. paramamosain*) were obtained from Zhangpu Fish Farm (Fujian, China) and were allowed to acclimate for 3 days before the experiments. *Staphylococcus aureus* [China General Microbiological Culture Collection Center (CGMCC) no. 1.2465], *Staphylococcus epidermidis* (CGMCC no. 1.4260), *Bacillus subtilis* (CGMCC no. 1.3358), *Listeria monocytogenes* (CGMCC no. 1.10753), *Pseudomonas stutzeri* (CGMCC no. 1.1803), *Acinetobacter baumannii* (CGMCC no. 1.6769), *Pseudomonas aeruginosa* (CGMCC no. 1.2421), *Pseudomonas fluorescens* (CGMCC no. 1.3202), *Escherichia coli* (CGMCC no. 1.2389), and *Vibrio alginolyticus* (CGMCC no. 1.1833) were purchased from CGMCC. In addition, the clinical isolates including MRSA QZ19131, MRSA QZ19132, MRSA QZ19133, MRSA QZ19134, MDR *P. aeruginosa* QZ18071, MDR *P. aeruginosa* QZ18072, MDR *P. aeruginosa* QZ18076, and MDR *P. aeruginosa* QZ18077 were provided by the Second Affiliated Hospital of Fujian Medical University (Quanzhou, Fujian, China). *Vibrio* was cultured in marine broth 2216 medium agar (BD DIFCO, USA) at 28°C, and the other strains were cultured in nutrient broth medium (OXBID, UK) at 37°C. The bacteria were washed in 10 mM sodium phosphate buffer (NaPB, pH 7.4) and diluted to the desired inoculum concentration according to the optical density at 600 nm. All experiments were carried out in strict accordance with the guidelines of Xiamen University.

### Cloning of full-length cDNA

According to the manufacturer’s instructions, the total RNA of gills from normal mature crabs was extracted using TRIzol™ reagent (Invitrogen, USA), and cDNA was generated using a PrimeScript™ RT reagent kit with a gDNA Eraser Kit (Takara, China). In addition, the cDNA templates for 5′- and 3′- random amplification of cDNA ends (RACE) PCR were synthesized using a SMARTer^®^ RACE 5′/3′ Kit (Takara, China). Gene-specific primers were designed to amplify the target gene based on the partial cDNA sequence obtained from the transcriptome database established in our laboratory (**Supplementary Table S1**). The amplified fragment was recombined into the pMD18-T vector (Takara, China) and sequenced by bioray biotechnology (Xiamen, China).

### Quantitative real-time PCR

Total RNA was extracted, and cDNA was generated as described above. Quantitative real-time PCR (qPCR) was performed on a Rotor-Gene Q (Qiagen, Germany) using FastStart DNA Master SYBR Green I (Roche Diagnostics). The tissue distribution of the Spgillcin transcripts was detected by absolute qPCR assay, and the immune responses of Spgillcin to different stimuli were measured by relative qPCR. *Sp-GAPDH* was chosen as the reference gene which was quantified to normalize *Spgillcin* gene expression. The specific primer sequences are listed in **Supplementary Table S1**, and the qPCR amplification conditions were set as follows: denaturing step at 95°C for 5 min and 30 cycles at 95°C for 30 s, 60°C for 30 s, and 72°C for 1 min.

### Sequence analysis, peptide synthesis, and antibiotics

The homology and similarity of the Spgillcin sequence was performed using the NCBI website (http://www.ncbi.nlm.nih.gov). The signal peptide of Spgillcin was predicted with SignalP 5.0 Server (http://www.cbs.dtu.dk/services/SignalP/), and the functional domain was predicted using SMART (http://smart.embl-heidelberg.de/). Network Protein Sequence@ website (http://www.prabi.fr) was used to predict the second structure of the Spgillcin mature peptide, and the physicochemical properties including molecular weight, hydrophobicity, net positive charge, and theoretical isoelectric point were predicted by ProtParam tool (https://web.expasy.org/protparam/).

The truncated peptide Spgillcin_177–189_ (KKRRCFFRHIYVA) was analyzed using antimicrobial peptide database CAMP_R.3_ (http://www.camp3.bicnirrh.res.in), and the physicochemical parameters of Spgillcin_177–189_ were predicted as mentioned above. This peptide was synthesized by Genscript (Naijing, China) and verified by high-performance liquid chromatography and mass spectrometry. AMP LL-37 was purchased from GL bioChem (Shanghai, China). The antibiotics including vancomycin, ceftazidime, ciprofloxacin, and polymyxin B were purchased from Solarbio company (Beijing, China), and rifampicin was purchased from Topscience company (Shanghai, China).

### Antimicrobial assay

The antimicrobial activity of Spgillcin_177–189_ was determined using the broth microdilution method as previously described (Shan et al., 2016). Briefly, a logarithmic-growth phase of bacteria was harvested and diluted in Muller-Hinton broth (HKM, China) to approximately 10^6^ colony-forming units (CFU) per milliliter, then added to wells, and incubated with different concentrations of peptides (1.5 to 96 μM) or different concentrations of antibiotics (0.01 to 4 μg ml^-1^) in 96-well polystyrene flat-bottomed plates (NEST, China). As the control, the bacteria were incubated with Milli-Q water. The microplates were subjected to static incubation at 37°C for 24 h. The minimal inhibitory concentration (MIC) values were defined as the lowest concentration without visible bacteria growth, and the minimal bactericidal concentration (MBC) values were determined by the minimum peptide concentration that killed ≥99.9% of bacteria. The experiments were performed in triplicate.

### The time killing kinetics

*S. aureus* (CGMCC no. 1.2465) and *P. aeruginosa* (CGMCC no. 1.2421) were chosen for time-killing studies as previously described (Shan et al., 2016). Briefly, a logarithmic-growth phase of bacteria was harvested and diluted in MH medium to approximately 10^6^ CFU ml^-1^, then 50 μl of suspension was added to wells, and these were incubated with 50 μl of Spgillcin_177–189_ (1× MIC). The cultures were sampled and plated onto NB agar plates at various time points. Then, the plates were incubated at 37°C for 18–24 h, and finally the number of viable counts was quantified. In the control group, these were cultured in the medium without peptide. Killing efficacy was calculated using the survival rate of colony-forming unit (%CFU) as follows: %CFU = recovered CFU/initial CFU × 100%, where initial CFU represented viable counts at 0 min and recovered CFU meant viable counts at different sampling points. The assays were performed in triplicate.

### Cytotoxicity assay

The cytotoxicity of Spgillcin_177–189_ to mammalian cell lines was evaluated using MTS assay as previously reported (Yang et al., 2020). Briefly, human hepatic cell line (L02), mouse liver cell line (AML12), hepatocellular carcinoma cell line (Hep G2), and non-small-cell lung carcinoma (NSCLC) cell line (NCI-H460) were provided by Stem Cell Bank, Chinese Academy of Sciences. L02 cells were cultured in RPMI-1640 medium (Invitrogen, USA) supplemented with 10% fetal bovine serum (FBS) (Gibco, Australia), and AML12 cells were cultured in Dulbecco’s modified Eagle’s medium/Nutrient Mixture F-12 supplemented with 10% FBS, 10 μg ml^-1^ human insulin, 5.5 μg ml^-1^ human transferrin, 5 ng ml^-1^ sodium selenite, and 40 ng ml^-1^ dexamethasone. Hep G2 cells were cultured in Dulbecco’s modified Eagle’s medium/Nutrient Mixture F-12 supplemented with 10% FBS. NCI-H460 cells were cultured in RPMI-1640 medium supplemented with 10% FBS. These cells were seeded into a 96-well cell culture plate (Thermo Fisher, USA), with a final cell density of approximately 1 × 10^5^ cells ml^-1^, and incubated overnight in an incubator with 5% CO_2_ atmosphere at 37°C. When these cells had adhered to the plate, the medium was removed, and these cells were cultured in fresh medium supplemented with various concentrations of peptide (0, 6, 12, 24, 48, and 96 μM). After an additional 24 h of incubation, cell viability was finally assessed using a CellTiter 96^®^ AQueous kit (Promega, USA). The experiments were carried out in triplicate.

### Thermal stability and cationic ion assays

The thermal activity of Spgillcin_177–189_ against *S. aureus* and *P. aeruginosa* was evaluated based on a previous report (Yu et al., 2022) with some modifications. Briefly, a logarithmic-growth phase of *S. aureus* and *P. aeruginosa* was harvested and diluted to approximately 1 × 10^6^ CFU ml^-1^. Spgillcin_177–189_ was subjected to a temperature of 100°C level for 10, 20, and 30 min, respectively, and then bacteria from different groups were prepared to be added to the wells and incubated with Spgillcin_177–189_. The microplate was incubated overnight at 37°C, and the absorbance was measured at 600 nm using a microplate reader (Tecan, Switzerland). For the cationic ion assay, the prepared bacteria were added to the wells and incubated with Spgillcin_177–189_ supplemented with different concentrations of NaCl salt solutions (Na^+^ levels from 10 to 160 mM). The microplate was incubated overnight at 37°C, and the absorbance was measured at 600 nm using a microplate reader (Tecan, Switzerland). The experiments were carried out in triplicate.

### Scanning electron microscope analysis

The effect of Spgillcin_177–189_ on *S. aureus* and *P. aeruginosa* was observed using SEM based on a previous report (Liu et al., 2020). *S. aureus* and *P. aeruginosa* were harvested in logarithmic-growth phase, resuspended in NaPB to approximately 1 × 10^7^ CFU ml^-1^, and then incubated with Spgillcin_177–189_ (1 × MIC) at room temperature for 30 min. After incubation, the samples were fixed in 2.5% (vol/vol) glutaraldehyde at 4°C overnight; then, the cells were washed three times, resuspended in about 10 μl of NaPB, and deposited on poly-L-lysine-coated glass slides at 4°C for 30 min. Afterwards, the cells were dehydrated using graded ethanol series (30, 50, 70, 90, 95, and 100%) for 15 min each. The samples were then dehydrated in a critical point dryer (EM CPD300, Leica, Germany). Finally, the specimens were coated with gold and then examined using a scanning electron microscope (Zeiss SUPRA 55, Germany).

### Transmission electron microscope analysis

The TEM analysis was carried out based on a previous report (Zhu et al., 2021) with slight modifications. Briefly, *S. aureus* and *P. aeruginosa* were prepared and incubated with Spgillcin_177–189_ as described above for the SEM analysis. After incubation, the cells were washed with NaPB three times and added to the agar models. Then, rice-grain-sized samples were fixed with 2.5% glutaraldehyde at 4°C overnight. After washing three times with NaPB for 15 min each, the samples were post-fixed with 1% osmium tetroxide, then dehydrated with gradient ethanol series, strained with uranyl acetate, rinsed in acetone, and embedded in epoxy resin. Finally, the samples were observed using a transmission electron microscope (HT7800, Hitachi, Japan).

### LTA and LPS inhibition assays

The effect of LTA and LPS on the bactericidal activity of Spgillcin_177–189_ was evaluated as described previously (Song et al., 2020) with slight modifications. Briefly, 25 μl of LTA (4 to 128 μg ml^-1^) from *S. aureus* (Sigma, USA) or 25 μl of LPS (4 to 128 μg ml^-1^) from *P. aeruginosa* (Sigma, USA) was added to a 96-well polypropylene flat-bottomed plate containing 25 μl of Spgillcin_177–189_ (1×, 2×, and 4× MIC) at room temperature for 30 min. Subsequently, 50 μl of *S. aureus* (1 × 10^6^ CFU ml^-1^) cells was added to the wells to incubate with the Spgillcin_177–189_–LTA mixture, or 50 μl of *P. aeruginosa* (1 × 10^6^ CFU ml^-1^) cells was added to the wells to incubate with the Spgillcin_177–189_–LPS mixture, respectively. After 18–24 h of incubation at 37°C, the MICs were measured at the absorbance of 595 nm using a microplate reader (Tecan Switzerland). The experiments were carried out in triplicate.

### Membrane permeability assay

The effect of peptide on membrane permeability was evaluated using LIVE/DEAD *Bac*Light™ bacterial viability kits (Thermo Fisher, USA) according to the manufacturer’s instructions. Briefly, *S. aureus* and *P. aeruginosa* in the logarithmic phase were harvested, and the cells were washed three times and resuspended in NaPB to a final density of approximately 1 × 10^7^ CFU ml^-1^. The prepared bacteria were then added to the wells to incubate with Spgillcin_177–189_ (1 × MIC) at 37°C for 30 min. After incubation, the bacteria were harvested and washed twice. SYTO 9 and PI were added to each sample according to the instructions, and the samples were incubated at room temperature in the dark for 15 min. Finally, fluorescent images were obtained using confocal laser scanning microscopy (Zeiss, Germany). Meanwhile, the samples were analyzed immediately by flow cytometry (CytoFLEX, Beckman, USA).

### ROS measurement

The levels of reactive oxygen species (ROS) in *S. aureus* and *P. aeruginosa* treated with Spgillcin_177–189_ or LL-37 were detected as described previously (Bai et al., 2021). Fluorescent probe 2′,7′-dichlorofluorescein diacetate (DCFH-DA) was used according to the manufacturer’s instructions (R&D, USA). Briefly, *S. aureus* and *P. aeruginosa* were cultured overnight in NB medium at 37°C. Then, the bacteria were washed three times and resuspended in NaPB. The suspension was diluted to approximately 1 × 10^7^ CFU ml^-1^ supplemented with 10 μM (final concentration) of DCFH-DA. Thereafter, 50 μl of the mixture was transferred into a 96-well flat-bottomed plate and incubated with 50 μl of Spgillcin_177–189_ (1 × MIC) or LL-37 (1 × MIC) in the dark at 37°C for 30 min. After incubation, fluorescence intensity was measured immediately using flow cytometry (CytoFLEX, Beckman, USA) with excitation wavelength at 488 nm and emission wavelength at 533 nm.

### Resistance development assay

The resistance of MRSA QZ19134 and MDR *P. aeruginosa* QZ18076 to Spgillcin_177–189_ was evaluated based on previous methods (Zhu et al., 2021) with slight modifications. Briefly, MRSA QZ19134 and MDR *P. aeruginosa* QZ18076 were harvested in logarithmic phase and diluted to approximately 1 × 10^6^ CFU ml^-1^. Then, 50 μl of bacterial suspension was added in triplicate to the wells of a 96-well polypropylene flat-bottomed plate containing 50 μl of Spgillcin_177–189_, LL-37, or antibiotics, including rifampicin, vancomycin, ceftazidime, and polymyxin B. The bacteria were incubated with Milli-Q water as the control group. The plates were subjected to static incubation at 37°C for 18–24 h, and the MIC of this day was defined as the lowest concentration of antimicrobial compound without visible bacterial growth. Thereafter, the cultures grown in 0.5-fold MIC suspension were diluted 1,000-fold to a new 96-well plate supplemented with different concentrations of peptides/antibiotics. The mixtures were incubated as described above and repeated after 50 days. The resistance evaluation of *S. aureus* (CGMCC no. 1.2465) and *P. aeruginosa* (CGMCC no. 1.2421) to Spgillcin_177–189_ adopted the same method.

### Biofilm inhibition assay

The inhibition effect of Spgillcin_177–189_ on biofilm formation was evaluated as described previously (de Breij et al., 2018; Yang et al., 2020). Briefly, *S. aureus* and *P. aeruginosa* cells were harvested in the logarithmic phase and resuspended in NaPB, washed twice and resuspended in MH medium, and then 50 μl of bacterial suspension was added to the wells and incubated with 50 μl of Spgillcin_177–189_ (0.75, 1.5, 3, and 6 μM). The microplate was statically incubated at 37°C for 24 h. After incubation, the biofilms were stained with 0.1% crystal violet, and the biofilm mass was measured at the absorbance of 595 nm using a microplate reader (Tecan, Switzerland). The experiments were carried out in triplicate.

### Antimicrobial assays in RAW 264.7 cells

The capacity of Spgillcin_177–189_ to eliminate extracellular *S. aureus* in the supernatant of RAW 264.7 cells was evaluated based on a previous description (Bai et al., 2021) with some modifications. Briefly, RAW 264.7 cells were diluted to a final cell density of 1 × 10^5^ cells ml^-1^, seeded in a 48-well cell culture plate (Thermo Fisher, USA), and then cultured in DMEM supplemented with 10% FBS in an incubator with 5% CO_2_ atmosphere at 37°C. When the cells had adhered to the plate, the DMEM was removed, and fresh complete medium (DMEM + 10% FBS) containing 10^4^ CFUs of *S. aureus* was added to the wells supplemented with three concentrations (4×, 8×, and 16× MIC) of Spgillcin_177–189_ or rifampicin or vancomycin, respectively. The cells were cultured in complete medium containing bacteria as the control group. The mixtures were incubated at 37°C for an additional 24 h. After incubation, the supernatants (5 μl) were serially diluted and transferred onto NB plates, and the CFUs were counted after incubation overnight at 37°C.

### Statistical analysis

All data were presented as mean ± standard deviations (SD). For the cytotoxicity assay, differences among groups were evaluated by one-way analysis of variance using SPSS Statistics 20 software. Statistical analysis was performed using GraphPad Prism 8.0 software, and differences were considered significant at *p*-value <0.05.

## Results

### The truncated peptide derived from novel protein Spgillcin

**Figure 1:**
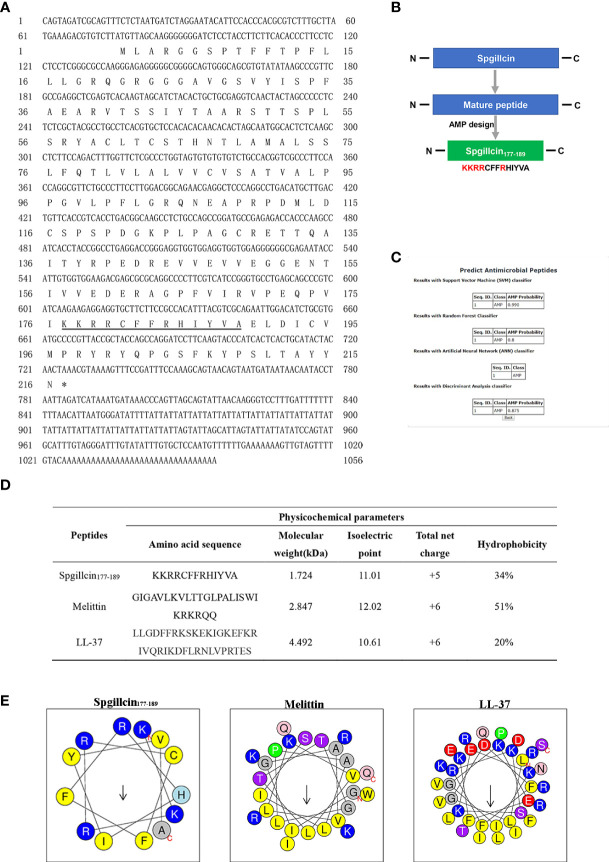
Sequence information and truncated peptide analysis. **(A)** Full-length cDNA and the deduced amino acid sequences of Spgillcin. The cDNA sequence had been deposited in Genbank, and the accession number was MZ131629. The underlined regions represent the amino acid sequence of Spgillcin_177–189_. **(B)** The design process of the truncated peptide and the cationic residues are marked in red. **(C)** The probability of Spgillcin_177–189_ to be a novel antimicrobial peptide (AMP) was calculated by four algorithms using AMP website database (CAMP_R.3_). **(D)** The key physicochemical parameters, Melittin and LL-37, were chosen as comparative controls. **(E)** Amino acid sequence helix diagrams (http://heliquest.ipmc.cnrs.fr) of Spgillcin_177–189_, Melittin, and LL-37.

The full-length cDNA sequence of Spgillcin was obtained, and the Genbank accession number was MZ131629. The novel gene was 1,056 bp, containing a 5′ untranslated region (UTR) of 75 bp, an open reading frame of 651 bp, and a 3′ UTR of 330 bp (**Figure 1A**). Spgillcin encoded 216 amino acids, and its calculated molecular weight was 23.615 kDa, with an estimated isoelectric point (PI) of 8.70. The total net charge of Spgillcin was +4, suggesting that it was a cationic protein. As shown in **Figure 1B**, the truncated peptide Spgillcin_177–189_ was derived from Spgillcin, which was located in 177th to 189th amino acid of the mature peptide. Additionally, based on a specific AMP database (CAMP_R.3_), Spgillcin_177–189_ was predicted to be a novel AMP candidate (**Figure 1C** **)**. The key physicochemical parameters of Spgillcin_177–189_ are shown in **Figures 1D, E**, with a comparative analysis using two known AMPs of Melittin and LL-37 as controls. The hydrophobicity of the three peptides ranged from 20 to 51%, the net charge ranged from + 5 to + 6, and the amino acid length was less than 50 residues. Collectively, these data indicated that Spgillcin_177–189_ satisfied the basic properties of known AMPs.

### Spgillcin exerts immune response *in vivo*

**Figure 2:**
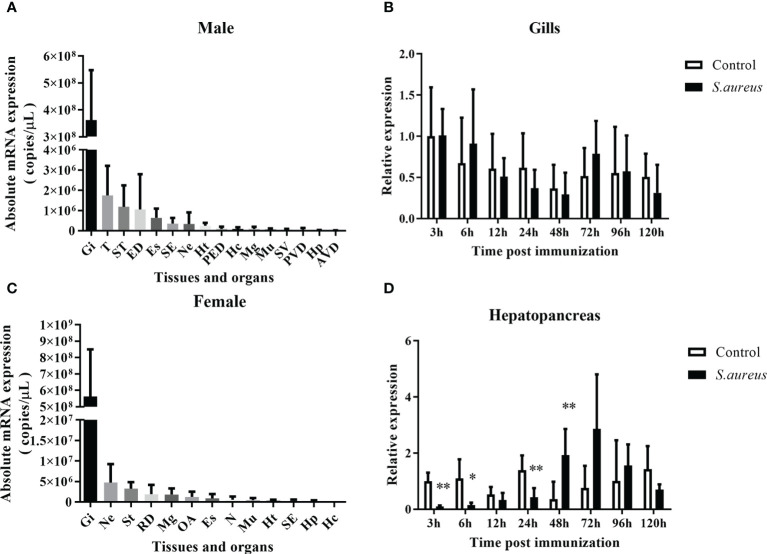
Expression profiles of Spgillcin in *S. paramamosain*. Tissue distribution of Spgillcin was detected in adult male **(A)** and female **(B)** crabs (*n* = 3). Relative expression levels of Spgillcin in the gills **(C)** and hepatopancreas **(D)** of male crabs after *S. aureus* challenge. Data are presented as mean ± standard deviation (SD), *P <0.05 and **P <0.01. Gi, gills; T, testis; ST, stomach; ED, ejaculatory duct; Es, eye stalk; SE, subcuticular epidermis; Ne, thoracic ganglion; Ht, heart; PED, posterior ejaculatory; Mg, midgut; Mu, muscle; SV, seminal vesicle; PVD, posterior vas deferens; RD, reproductive duct; Hp, hepatopancreas; AVD, anterior vas deferens; OA, ovary; Hc, hemocytes; N, spermathecae.

The transcriptional pattern of Spgillcin was tested in various tissues. As shown in **Figures 2A, B**, the expression levels of Spgillcin were varied and dominantly expressed in the gills of male and female adult crabs. Thus, the expression profile in the gills of male crabs with bacterial challenge was investigated. As shown in **Figure 2C**, Spgillcin expression was not significantly induced after *S. aureus* challenge at detecting time-point (6, 12, 24, 48, 96, and 120 h), whereas the expression level of Spgillcin in the hepatopancreas had a significant downregulation at 3, 6, and 24 h and upregulation at 48 h (**Figure 2D**). We also tested the expression level of Spgillcin at the megalops stage. As shown in **Supplementary Figures S1A, B**, the expression level of Spgillcin had significant downregulation and upregulation after the LPS and *V. alginolyticus* challenge, respectively.

### Spgillcin_177–189_ exerts potent broad-spectrum antimicrobial activity

The antimicrobial and bactericidal activities of Spgillcin_177–189_ against a series of strains were examined. As shown in **Table 1**, the data showed that Spgillcin_177–189_ exhibited a broad-spectrum antimicrobial activity against Gram-positive (*S. epidermidis*, *S. aureus*, *B. subtilis*, and *L. monocytogenes*) and Gram-negative (*P. stutzeri*, *P. aeruginosa*, *A. baumannii*, *P. fluorescens*, and *E. coli*) bacteria with MIC values from 1.5 to 48 μM and MBC values lower than 48 μM. In addition, the antimicrobial effect of Spgillcin_177–189_ against clinical isolates (MRSA and MDR *P. aeruginosa*) was also investigated. Spgillcin_177–189_ exhibited a potent antimicrobial activity against MRSA and MDR *P. aeruginosa* with MIC values from 6 to 24 μM and MBC values below 48 μM.

**Table 1:** Antimicrobial activity of Spgillcin_177–189_ against microorganisms.

Microorganisms	CGMCC number^a^	Spgillcin_177–189_	
		MIC^b^	MBC^c^
Gram-positive bacteria			
*Staphylococcus aureus*	1.2465	6–12	12
*Staphylococcus epidermidis*	1.4260	3–6	6
*Bacillus subtilis*	1.3358	12–24	24
*Listeria monocytogenes*	1.10753	24–48	48
Gram-negative bacteria			
*Pseudomonas fluorescens*	1.3202	6–12	12
*Acinetobacter baumannii*	1.6769	12–24	24
*Pseudomonas aeruginosa*	1.2421	6–12	12
*Pseudomonas stutzeri*	1.1803	1.5–3	3
*Escherichia coli*	1.2389	12–24	24
Clinical isolates			
MRSA QZ19131	–	6–12	12
MRSA QZ19132	–	6–12	12
MRSA QZ19133	–	6–12	12
MRSA QZ19134	–	6–12	12
MDR *P. aeruginosa* QZ18071	–	12–24	24
MDR *P. aeruginosa* QZ18072	–	12–24	48
MDR *P. aeruginosa* QZ18076	–	12–24	24
MDR *P. aeruginosa* QZ18077	–	12–24	48

a China General Microbiological Culture Collection Center.

b The values of MIC were expressed as the interval [a]-[b]. [a] was the highest concentration tested with visible microbial growth, whereas [b] was determined as the lowest concentration without visible microbial growth.

c The values of MBC presented are those wherein the peptide concentration killed 99.99% of the bacteria.

### Spgillcin_177–189_ exerts rapid bactericidal kinetics

**Figure 3:**
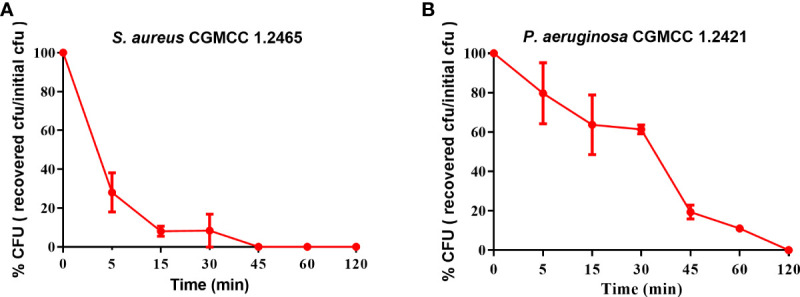
Time-killing curves of Spgillcin_177–189_ on *S. aureus* and *P. aeruginosa*. **(A)** Time-killing curve of Spgillcin_177–189_ on *S. aureus*. **(B)** Time-killing curve of Spgillcin_177–189_ on *P. aeruginosa*.

The bactericidal efficacy of Spgillcin_177–189_ was further evaluated. *S. aureus* (CGMCC 1.2645) and *P. aeruginosa* (CGMCC 1.2421) were chosen for time-killing kinetic curves. As shown in **Figure 3A**, Spgillcin_177–189_ killed 99.9% of *S. aureus* in approximately 45 min. Meanwhile, Spgillcin_177–189_ had a potent bactericidal efficacy with a killing rate of 99.9% against *P. aeruginosa* within 120 min (**Figure 3B**).

### Spgillcin_177–189_ shows no cytotoxicity to mammalian cell lines

**Figure 4:**
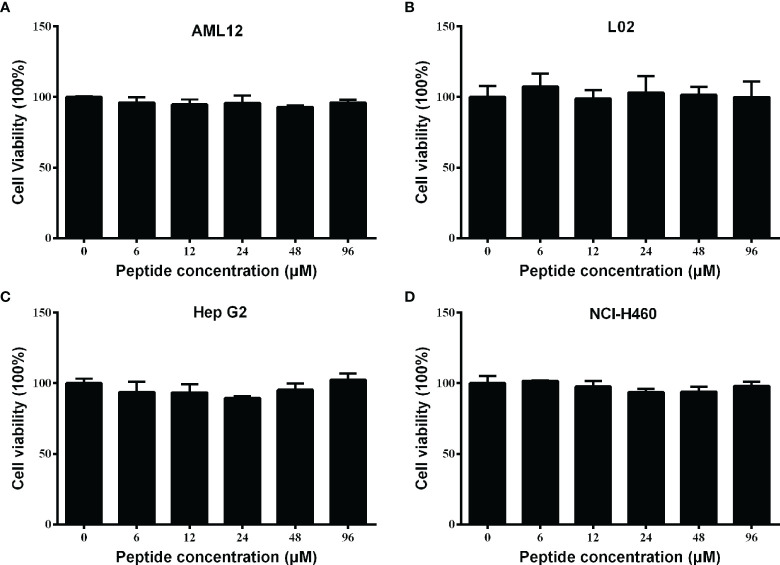
Cytotoxic effect of Spgillcin_177–189_ towards mammalian cell lines. **(A)** Cytotoxicity of Spgillcin_177–189_ on murine hepatic cell line AML 12. **(B)** Cytotoxicity of Spgillcin_177–189_ on human hepatic cell line L02. **(C)** Anti-cancer activity of Spgillcin_177–189_ on hepatocellular carcinoma cell line Hep G2. **(D)** Anti-cancer activity of Spgillcin_177–189_ on NSCLC cell line NCI-H460. These cells were cultured in complete medium at various concentrations of Spgillcin_177–189_. Cell viability was measured by MTS method, and data represent the mean ± SD from three independent biological replicates.

For future application, it is necessary to assess the safety of Spgillcin_177–189_. The cell viability of AML 12, L02, Hep G2, and NCI-H460 cells after Spgillcin_177–189_ treatment was evaluated using MTS assay. As shown in **Figures 4A, B**, Spgillcin_177–189_ showed no cytotoxic effect on AML 12 and L02 cell growth at concentrations ranging from 3 to 96 μM. In addition, the anti-cancer activity of Spgillcin_177–189_ was investigated, as shown in **Figures 4C** **, D**. The quantities of Hep G2 and NCI-H460 cells did not significantly decrease after the Spgillcin_177–189_ treatment (3–96 μM), indicating that Spgillcin_177–189_ may not be an anti-tumor agent.

### The stability and antimicrobial activity of Spgillcin_177–189_

**Figure 5:**
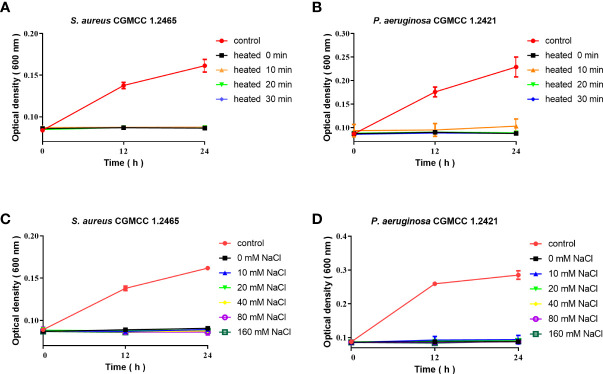
Thermal stability and cationic ion activity of Spgillcin_177–189_ against *S. aureus* and *P. aeruginosa*. **(A)** Thermal stability of Spgillcin_177–189_ against *S. aureus.* **(B)** Thermal stability of Spgillcin_177–189_ against *P. aeruginosa*. **(C)** Antimicrobial activity of Spgillcin_177–189_ against *S. aureus* at different concentrations of NaCl salt solutions. **(D)** Antimicrobial activity of Spgillcin_177–189_ against *P. aeruginosa* at different concentrations of NaCl salt solutions.

The antimicrobial activity of Spgillcin_177–189_ was evaluated under different incubation conditions. As shown in **Figures 5A, B**, Spgillcin_177–189_ maintained its bactericidal activity against *S. aureus* and *P. aeruginosa* even when the peptide was subjected to a high temperature of 100°C for 10, 20, and 30 min. This result better characterized the thermal stability of Spgillcin_177–189_. In addition, the effect of cation on bacterial sensitivity was assessed as shown in **Figures 5C** **, D**. *S. aureus* and *P. aeruginosa* remained sensitive to Spgillcin_177–189_ in NaCl solutions ranging from 10 to 160 mM, indicating that the Na^+^ levels did not affect the bacterial sensitivity to Spgillcin_177–189_.

### The morphological changes of *S. aureus* and *P. aeruginosa* after Spgillcin_177–189_ treatment

**Figure 6:**
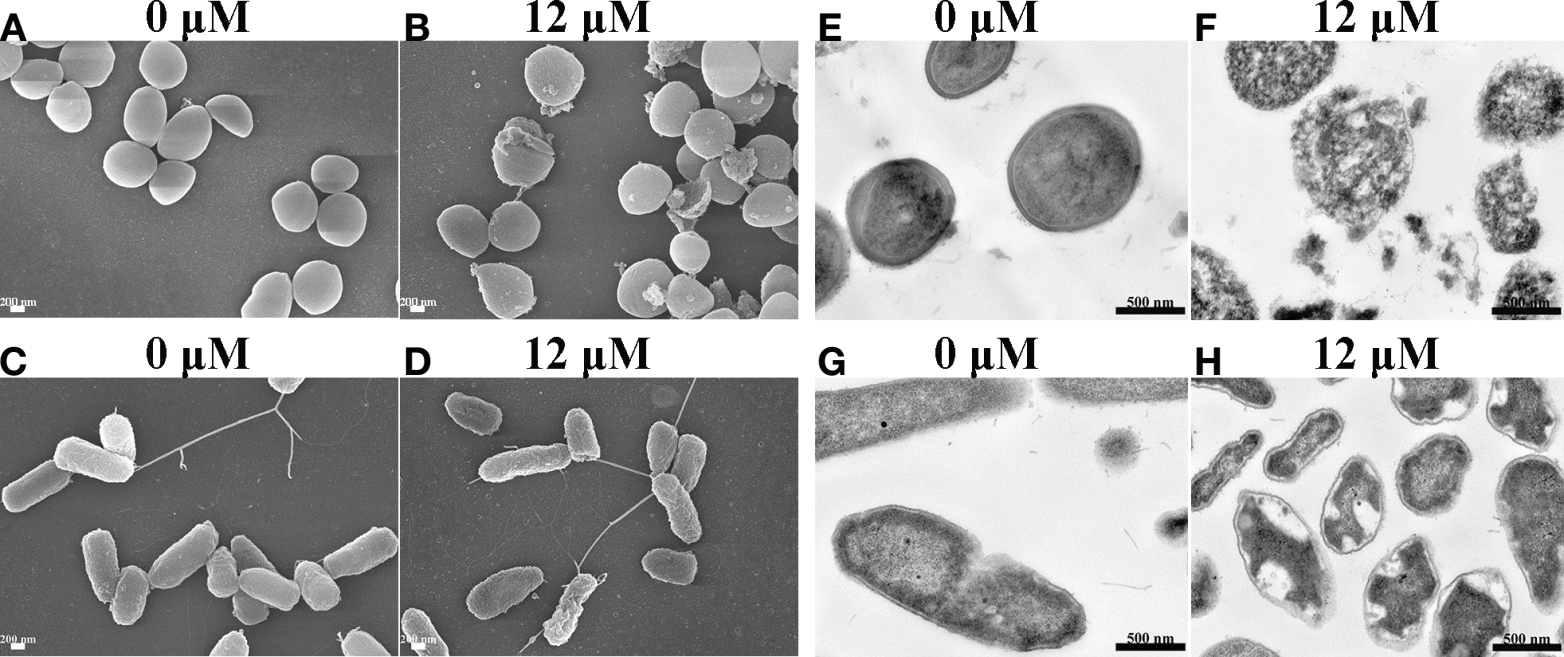
Electron micrographs of *S. aureus* and *P. aeruginosa*. Scanning electron micrographs of *S. aureus* treated with NaPB **(A)** or Spgillcin_177–189_ **(B)**; scale bars, 200 nM. Scanning electron micrographs of *P. aeruginosa* treated with NaPB **(C)** or Spgillcin_177–189_ **(D)**; scale bars, 200 nM. Transmission electron micrographs of *S. aureus* treated with NaPB **(E)** or Spgillcin_177–189_ **(F)**; scale bars, 500 nM. Transmission electron micrographs of *P. aeruginosa* treated with NaPB **(G)** or Spgillcin_177–189_ **(H)**; scale bars, 500 nM.

To uncover the antimicrobial mechanism of Spgillcin_177–189_, the morphological changes of *S. aureus* and *P. aeruginosa* after Spgillcin_177–189_ treatment were observed by using SEM. As shown in **Figure 6A**, the bacteria showed a smooth surface morphology in the control group, whereas the membrane integrity of *S. aureus* was significantly disrupted after exposure to Spgillcin_177–189_ (**Figure 6B** **)**. Meanwhile, without treatment, the *P. aeruginosa* cell membranes were of intact integrity (**Figure 6C**), whereas *P. aeruginosa* cells showed a wrinkled membrane surface after Spgillcin_177–189_ treatment as shown in **Figure 6D**. To visualize the membrane structure and cytoplasmic changes, TEM was used to observe the thin sections of *S. aureus* and *P. aeruginosa* cells after exposure to Spgillcin_177–189_, as shown in **Figure 6E**. In the control group, *S. aureus* cells showed an intact double-membrane cell envelope structure. However, after treatment with Spgillcin_177–189_, the membrane structure was significantly ruptured, and there was leakage of intracellular contents as well (**Figure 6F** **)**. In addition, the membrane structure of *P. aeruginosa* cells was clearly visible in **Figure 6G**. After treatment with Spgillcin_177–189_, the *P. aeruginosa* cells showed a shrinkage phenomenon of their inner membrane (**Figure 6H**). These data indicated that Spgillcin_177–189_ increased the membrane permeability and caused bacterial membrane damage and leakage of the cytoplasm.

### Bacterial membrane component inhibits the antimicrobial activity of Spgillcin_177–189_

**Figure 7:**
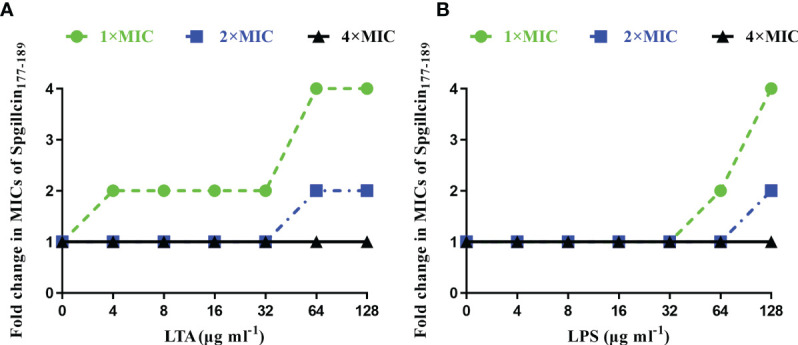
Inhibitory effect of lipoteichoic acid (LTA) and lipopolysaccharide (LPS). **(A)** LTA impaired the bactericidal activity of Spgillcin_177–189_ against *S. aureus* in a dose-dependent manner. **(B)** LPS impaired the bactericidal activity of Spgillcin_177–189_ against *P*. *aeruginosa* in a dose-dependent manner.

In most cases, to kill bacteria, AMPs first bind to the surface of bacterial membranes. LTA and LPS are key bacterial membrane components of Gram-positive and Gram-negative bacteria, respectively. AMPs can interact with LTA and LPS to exhibit broad-spectrum antimicrobial effects. Therefore, the antimicrobial activity of Spgillcin_177–189_ against *S. aureus* after the addition of LTA and against *P. aeruginosa* after the addition of LPS was evaluated, respectively. As shown in **Figure 7A**, without an exogenous addition of LTA, the antimicrobial activity of Spgillcin_177–189_ against *S. aureus* remained the same, whereas the MIC value increased twofold after an exogenous addition of LTA (4, 8, 16, and 32 μg ml^-1^) and increased fourfold after the addition of LTA in the range of 64 to 128 μg ml^-1^. The antimicrobial activity of Spgillcin_177–189_ against *P. aeruginosa* with exposure to LPS was also evaluated, as shown in **Figure 7B**. The MIC value of Spgillcin_177–189_ against *P. aeruginosa* was unchanged without and with low concentrations of LPS (4, 8, 16, and 32 μg ml^-1^), but when LPS was exogenously added at levels of 64 and 128 μg ml^-1^, the MIC values increased two- and fourfold, respectively. These results illustrated that LTA and LPS inhibited the bactericidal efficacy of Spgillcin_177–189_ against bacteria in a dose-dependent manner.

### Spgillcin_177–189_ increases the bacterial membrane permeability of *S. aureus* and *P. aeruginosa*

**Figure 8:**
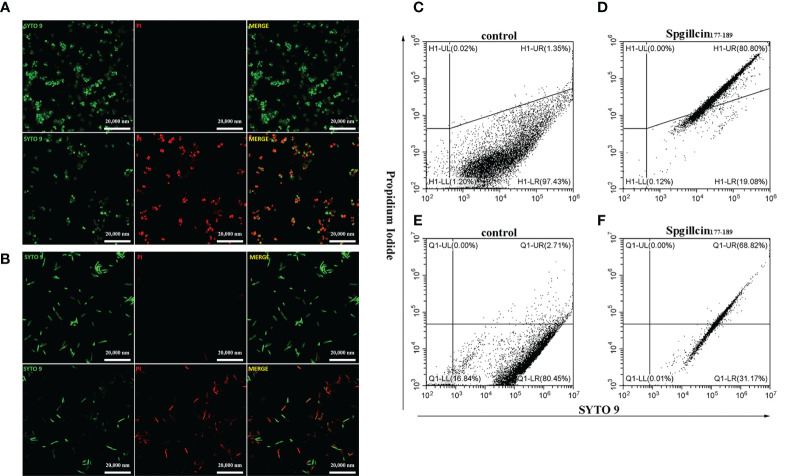
Effect of Spgillcin_177–189_ on the membrane permeability of *S. aureus* and *P. aeruginosa*. **(A)** *S. aureus* cells were resuspended in culture medium and treated with NaPB (control) or Spgillcin_177–189_ (1× minimum inhibitory concentration). Fluorescent images of stained bacteria were obtained using confocal scanning microcopy. The upper row was the control group, and the lower row was the Spgillcin_177–189_-treated group; scale bars, 20,000 nM. **(B)** *P. aeruginosa* cells were suspended in culture medium and treated with NaPB (control) or Spgillcin_177–189_ (1× minimum inhibitory concentration). Fluorescent images of stained bacteria were obtained using confocal scanning microcopy. The upper row was the control group, and the lower row was the Spgillcin_177–189_-treated group; scale bars, 20,000 nM. The membrane permeability of *S. aureus* exposed to NaPB **(C)** or Spgillcin_177–189_ **(D)** was examined by flow cytometry. The membrane permeability of *P. aeruginosa* exposed to NaPB **(E)** or Spgillcin_177–189_ **(F)** was examined by flow cytometry.

Microbial membranes act as a physical barrier to provide a protective function. The main mechanism of AMPs is targeting bacterial membrane and changing membrane permeability. To test the presumption, we examined the fluorescent intensity of *S. aureus* and *P. aeruginosa* using CLSM. As shown in **Figure 8A**, without treatment with Spgillcin_177–189_, the bacterial membrane of *S. aureus* was intact and PI fluorescence was not observed. Compared to the control, almost all the tested bacterial cells were labeled with PI signals. Meanwhile, we monitored the PI intensity of *P. aeruginosa* cells. PI fluorescence was not observed in the bacteria without treatment, whereas the *P. aeruginosa* cells appeared to have a remarkable PI fluorescence after exposure to Spgillcin_177–189_ (**Figure 8B**). This fact was verified again using flow cytometry. Compared to the control (**Figure 8C**), the number of *S. aureus* in the PI channel significantly increased after exposure to Spgillcin_177–189_ (**Figure 8D**). Meanwhile, we tested the PI signal intensity of *P. aeruginosa*. The *P. aeruginosa* cells failed to exert a PI uptake activity in the control group (**Figure 8E**). However, the number of PI-labeled *P. aeruginosa* significantly increased after treatment with Spgillcin_177–189_ (**Figure 8F**). Collectively, these data indicated that the integrity of the bacterial plasma membrane was damaged after the Spgillcin_177–189_ treatment.

### Spgillcin_177–189_ induces intracellular ROS levels

**Figure 9:**
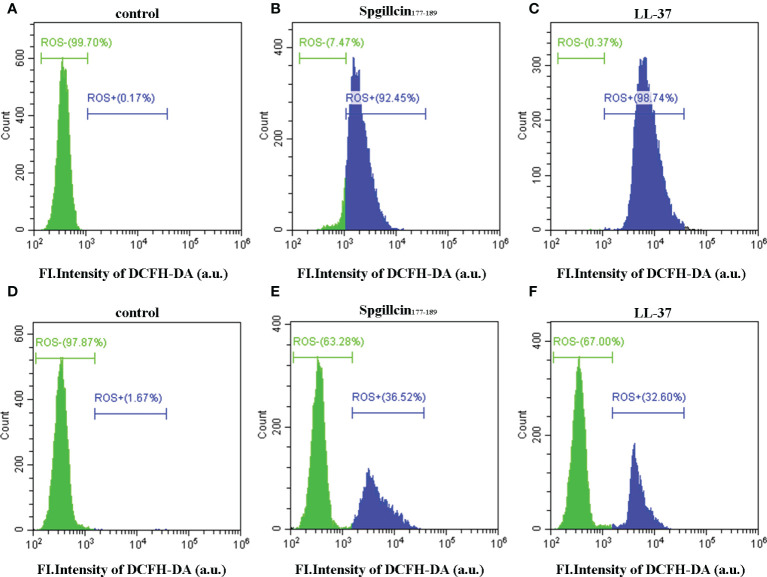
Detection of intracellular reactive oxygen species levels. Fluorescence intensity of DCFH-DA probe on *S. aureus* treated with NaPB **(A)**, Spgillcin_177–189_ **(B)**, or LL-37 **(C)**. Fluorescence intensity of DCFH-DA probe on *P. aeruginosa* treated with NaPB **(D)**, Spgillcin_177–189_ **(E)**, or LL-37 **(F)**.

Antimicrobial peptides usually induce ROS generation to aggravate membrane damage. We presumed that Spgillcin_177–189_ could increase the accumulation of ROS. To test this hypothesis, LL-37 was chosen as the positive control, and DCFH-DA probe was used to measure the intracellular ROS levels of *S. aureus* and *P. aeruginosa*. As shown in **Figure 9A**, without treatment, the bacteria did not generate ROS in the control group, whereas *S. aureus* cells significantly increased the ROS levels after exposure to Spgillcin_177–189_ (**Figure 9B**) or LL-37 (**Figure 9C**). Meanwhile, we measured the intracellular ROS levels of *P. aeruginosa*. Compared to the control (**Figure 9D**), *P. aeruginosa* cells significantly increased the ROS levels when treated with Spgillcin_177–189_ (**Figure 9E**) or LL-37 (**Figure 9F**). These data illustrated that Spgillcin_177–189_ could cause an accumulation of intracellular ROS in *S. aureus* and *P. aeruginosa*.

### Spgillcin_177–189_ kills MRSA QZ19134 and MDR *P. aeruginosa* QZ18076 without resistance selection

**Figure 10:**
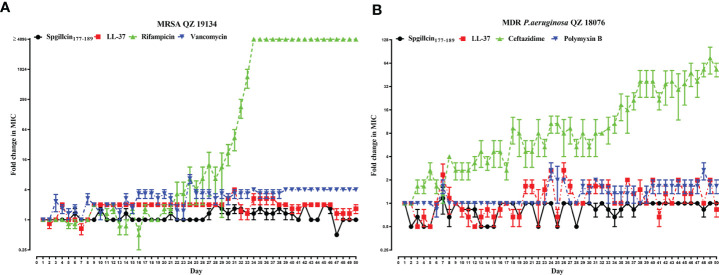
Spgillcin_177–189_ killed MRSA and MDR *P. aeruginosa* without resistance selection. **(A)** Resistance development of MRSA QZ19134 after continuous Spgillcin_177–189_, LL-37, rifampicin, and vancomycin treatment. **(B)** Resistance development of MDR *P. aeruginosa* after continuous Spgillcin_177–189_, LL-37, ceftazidime, and polymyxin B treatment. The y-axis values represented fold change (in log_2_) in minimum inhibitory concentration (MIC) relative to the MIC of the first day during serial passage, and the x-axis showed the number of days.

The lifespan of antibiotics depends on the drug resistance rate. AMPs targeting and physically disrupting bacterial membranes result in low resistance development. Therefore, we assessed the possibility of MRSA QZ19134 and MDR *P. aeruginosa* QZ18076 to develop resistance following the continuous Spgillcin_177–189_ treatment. In addition, LL-37 and two antibiotics were chosen for comparative analysis. As shown in **Figure 10A**, MRSA QZ19134 failed to produce resistant mutants under the treatment of Spgillcin_177–189_ in the presence of a sub-inhibitory concentration after a 50-day serial passage, and the MIC value was unchanged from the first day to the 50th day (the MIC value was 12 μM at the first and at the last day). For LL-37, the MIC value increased twofold after the 50-day consecutive passage (the MIC value was 12 μM at the first day and 24 μM at the 50th day). However, MRSA QZ19134 developed significant resistance after repeated treatment with the antibiotic rifampicin, and the MIC value had a >4,096-fold change (the MIC value was 0.03 μg ml^-1^ at the first day and was 1,024 μg ml^-1^ at the 50th day). In contrast, MRSA QZ19134 remained sensitive to vancomycin, with only a fourfold change in the MIC value after 50 days of successive culturing (the MIC value was 0.25 μg ml^-1^ at the first day and 1 μg ml^-1^ at the 50th day). Afterwards, we also evaluated the trend of drug resistance for MDR *P. aeruginosa* QZ18076. As shown in **Figure 10B**, Spgillcin_177–189_ killed MDR *P. aeruginosa* QZ18076 without resistance selection after the 50-day serial passage with no change in the MIC value (the MIC value was 24 μM at the first and at the last day). The MIC value of LL-37 did not increase from the first day to the 50th day, just remaining at 12 μM. However, ceftazidime led to a notable developed resistance in MDR *P. aeruginosa* QZ18076, with a 64-fold increase in the MIC value at the 50th day (the MIC value was 2 μg ml^-1^ at the first day and 128 μg ml^-1^ at the 50th day). Polymyxin B failed to produce drug resistance when MDR *P. aeruginosa* QZ18076 was serially passaged (the MIC value was 4 μg ml^-1^ at the first day and 8 μg ml^-1^ at the 50th day). Additionally, *S. aureus* (CGMCC 1.2465) did not produce resistant mutants after the 50-day serial passage (the MIC value was 12 μM at the first day and 24 μM at the 50th day). Meanwhile, the MIC value of Spgillcin_177–189_ against *P. aeruginosa* (CGMCC 1.2421) did not change from the first day to the 26th day (the MIC value was 12 μM at the first and at the last day) as shown in the **Supplementary Figures S2A, B**.

### Spgillcin_177–189_ has anti-biofilm activity against *S. aureus* and *P. aeruginosa*

**Figure 11:**
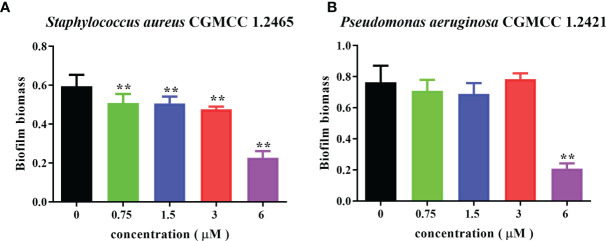
Inhibitory effect of Spgillcin_177–189_ on *S. aureus* and *P. aeruginosa* biofilm formation. **(A)** *S. aureus* and **(B)** *P. aeruginosa* were incubated without or with different concentrations of Spgillcin_177–189_. Biofilm mass was quantified by crystal violet staining, and then the absorbance was measured at 595 nm. Data represented mean ± standard error of mean, and the experiments were of three biological replicates completed independently. The significant difference between control group and Spgillcin_177–189_-treated group is indicated by asterisks as **p <0.01.

Bacterial biofilms can attenuate the antibiotics’ bactericidal effects. Spgillcin_177–189_ exhibits potent antimicrobial activity against *S. aureus* and *P. aeruginosa*. We further evaluated the ability of Spgillcin_177–189_ to prevent biofilm formation. As shown in **Figure 11A**, Spgillcin_177–189_ significantly inhibited *S. aureus* biofilm information at concentrations of 0.75, 1.5, 3, and 6 μM. Meanwhile, when *P. aeruginosa* cells were exposed to different concentrations of Spgillcin_177–189_, the concentration of Spgillcin_177–189_ required to inhibit biofilm formation was 6 μM (**Figure 11B**), indicating that Spgillcin_177–189_ exhibited admirable biofilm inhibition effects on *S. aureus* and *P. aeruginosa*.

### Spgillcin_177–189_ kills extracellular *S. aureus* in the presence of RAW 264.7 cells

**Figure 12:**
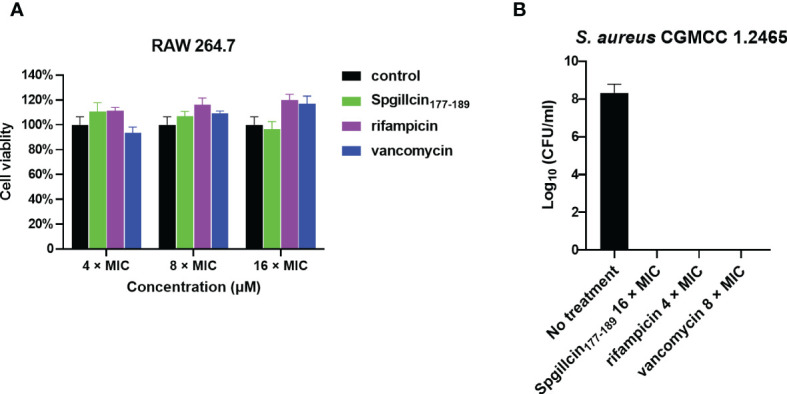
Spgillcin_177–189_ eradicated extracellular *S. aureus* in the supernatant of RAW 264.7 cells. **(A)** The cell viability of RAW 264.7 cells was evaluated after different concentrations [4×, 8×, and 16× minimum inhibitory concentration (MIC); the MIC values of rifampicin and vancomycin against *S. aureus* were 0.01 and 0.25 μg ml^-1^, respectively] of Spgillcin_177–189_ or rifampicin or vancomycin treatment. RAW 264.7 cells were cultured in a medium without peptide/antibiotics as control. **(B)** Bacteria burden determination of *S. aureus*-infected RAW 264.7 cells after 24 h of using Spgillcin_177–189_ (16× MIC) or rifampicin (4× MIC) or vancomycin (8× MIC) treatment.

Since Spgillcin_177–189_ displays potent antimicrobial and anti-biofilm activities against *S. aureus*, we further evaluated whether Spgillcin_177–189_ can eliminate *S. aureus* in the supernatant of RAW 264.7 cells. The antibiotics rifampicin and vancomycin were chosen for comparative analysis. RAW 264.7 cells showed no cytotoxic effect after Spgillcin_177–189_ or rifampicin or vancomycin treatment (**Figure 12A**). As shown in **Figure 12B**, when RAW 264.7 cells and *S. aureus* were cocultured in complete medium supplemented with Spgillcin_177–189_ or rifampicin or vancomycin after 24 h of infection, compared to the control, Spgillcin_177–189_ (16× MIC) killed all *S. aureus* in the supernatant of RAW 264.7 cells. Meanwhile, rifampicin (4× MIC) and vancomycin (8× MIC) eliminated extracellular *S. aureus* in the presence of RAW 264.7 cells, respectively. Thus, Spgillcin_177–189_ could achieve clearance of extracellular *S. aureus* in the supernatant of RAW 264.7 cells and excellent biocompatibility at a working concentration.

## Discussion

Over the past 80 years, antibiotics have made a great contribution to human and animal health by saving innumerable lives worldwide since their routine use in the 1940s (Browne et al., 2020). However, the emergence of bacterial multidrug resistance to antibiotics has gradually become a serious threat to public health, and this critical trend seems uncontrollable (O’Neill, 2016). AMPs are considered “natural antibiotics” and may have the most potential as substitutes for antibiotics that are seeing significant drug resistance. Thus, the exploration of new antimicrobial agents from a variety of species, including marine animals, attracts multidisciplinary scientists to this field. AMPs, as powerful alternatives, possess potent antibacterial effects and unique microbial membrane disruption mechanisms (Brogden, 2005; Magana et al., 2020), which are worthwhile to explore in living organisms in the ocean or on land. The priority is to discover novel genes with predicted antimicrobial activities, which is important because the number of known AMPs is not sufficient for their demand in medical fields. Marine animals are one of the optimal resources of AMPs due to their diverse morphologies and molecular structures that naturally provide plenty of distinctive AMPs (Hoang and Kim, 2013; Semreen et al., 2018). In this study, we screened one functional gene named *Spgillcin* from the transcriptomics data of *S. paramamosain* using bacterial challenge. A subsequent *in vivo* investigation indicates that Spgillcin is highly expressed in mud crab gills, which are usually described as immune organs and associated with innate immune defense. Interestingly, Spgillcin showed a significant response in the hepatopancreas rather than in the gills when the mud crab was challenged with *S. aureus*. A possible reason for the high expression of Spgillcin in gills is the daily maintenance of crab health, while the high expression in the hepatopancreas might be an anti-infection requirement. No matter the reason, the *in vivo* study demonstrated that Spgillcin is a functional protein, and its role is at least associated with the immune system defense against bacterial invasion.

Similar to some short peptides identified in our previous studies, we found a sequence of 13 amino acids in the truncated peptide Spgillcin_177–189_. Based on the sequence of Spgillcin, it is active against bacteria. Accordingly, the physicochemical property of Spgillcin_177–189_ was elucidated to be related to this activity. Among the reported synthetic peptides, some are directly derived from the genes activated *in vivo*, such as Spgillcin which was linked to mRNA expression and generated an immune response to the *in vivo* bacterial challenge*in vivo* of *S. paramamosain*. Conversely, some peptides are chemically synthesized or based on expressed sequence tag sequences released on Genbank. Both processes are universally recognized as ways to rationally design or modify parent peptide sequences to obtain antimicrobial agents and improve antimicrobial properties. Ω76 is an AMP which was designed based on α-helical AMP structures extracted from the APD database and displays *in vitro* and *in vivo* efficacy against carbapenem- and tigecyline-resistant *A*. *baumannii* (Nagarajan et al., 2019). SAAP-148, which is a synthetic derivative of the human antimicrobial peptide LL-37, significantly enhances antimicrobial activities under the same physiological conditions compared to its parent peptide (de Breij et al., 2018). AA139, an AMP found in marine species, displays a broad-spectrum activity against clinical Gram-positive and Gram-negative bacteria (Elliott et al., 2020). Spgillcin_177–189_ also has desirable properties by killing the clinical isolates of multidrug-resistant pathogens, which are particularly concerning to the public as they usually generate resistance to multiple antibiotics, thus severely restricting the clinical treatment options. In view of its efficacy on multidrug-resistant bacteria, Spgillcin_177–189_, the novel AMP identified in mud crabs, appears to be a candidate to be employed as a potential therapeutic substance in the future.

The characteristics of Spgillcin_177–189_ and the feasibility of its application to medicine were analyzed in this study. The stability of AMPs is a key concern and often becomes a constraint to extensive applications under complex conditions. In this study, Spgillcin_177–189_ maintained its antimicrobial activity and was stable even with changing temperature and salt conditions. A steady-state condition may be a prerequisite for the further application of Spgillcin_177–189_, as it has been reported previously that the AMP Microcin J25 can effectively kill MDR *E. coli* even during fluctuating pH and temperature (Yu et al., 2022). Cytotoxicity is also a primary drawback to the clinical application of peptide drugs (van der Kraan et al., 2004), also restricting the topical utilization of AMPs. Spgillcin_177–189_ showed no cytotoxicity to normal cells AML12 and L02 even at higher concentrations. Similar to some known AMPs, Spgillcin_177–189_ showed no anticancer activity to either the hepatoma cell line Hep G2 or the non-small lung carcinoma cell line NCI-H460 tested in this study. This feature differs among AMPs, as some possess both antibacterial and anticancer activities—for example, the host immune defense peptide LL-37 activates caspase-independent apoptosis and exercises the suppression of colon cancer (Ren et al., 2012), while a short-designed peptide, G3, possesses both antibacterial and anticancer activities (Hu et al., 2011; Chen et al., 2015). Our recent publication also showed that a novel AMP Scyreprocin, which is discovered in *S. paramamosain*, has strong activities against bacteria, fungi, and cancer (Yang et al., 2022). This study indicates that Spgillcin_177–189_ is not a promising candidate for cancer therapy in the future.

Now, it is clearly known that the principal antibacterial mechanism of most AMPs against bacteria was by disrupting their membranes, causing content leakage and ultimately resulting in bacteria death (Ma et al., 2016; Huo et al., 2020). Spgillcin_177–189_ displayed the same activity mode. Both SEM and TEM results indicated that the Spgillcin_177–189_ activity was mainly focused on cell membranes, damaging their integrity and resulting in the appearance of fragmentation and irregularity on the cell surface. In an earlier study, another AMP, EC1-17KV, also showed bactericidal activity against drug-resistant *P. aeruginosa* by destroying the cell membranes (Ma et al., 2020), although the source was different from Spgillcin_177–189_. Interestingly, the antibacterial mode of Spgillcin_177–189_ presented differently in *S. aureus* and *P. aeruginosa*, causing cell content leakage in *S. aureus* and complete destruction of the cellular structure in *P. aeruginosa*. A similar result was also observed in our previous study of SpHyastatin (Shan et al., 2016), which showed different antimicrobial mechanisms on *S. aureus* and *P*. *fluorescens*. The reason for this disparity might be related to differences in the composition of their bacterial cell walls. It is well known that LTA is a principle component of microbial cell walls in Gram-positive bacteria (Kondo et al., 2012), and LPS is an abundant component of bacterial outer membranes in Gram-negative bacteria (Simpson and Trent, 2019), the two types of bacteria possessing distinct membrane components that are targets for AMPs (Epand et al., 2016). Therefore, it is important to investigate if the antibacterial mechanism of Spgillcin_177–189_ proceeds differently on the bacterial membrane components of Gram-negative and Gram-positive bacteria. Our observations from adding different doses of LTA or LPS in chequerboard broth microdilution assays demonstrated the association between the antimicrobial activity of Spgillcin_177–189_ and the bacterial membrane components. This association is probably attributable to the electrostatic interactions because both LTA and LPS belong to negatively charged bacterial components. Cationic Spgillcin_177–189_ can target two components of likely LTA/LPS-targeting (Stokes et al., 2017; Devnarain et al., 2021) peptides. In addition, this study showed that the ROS levels of *S. aureus* and *P. aeruginosa* were increased after treatment with Spgillcin_177–189_, which may be related to the bacterial death of these strains. Similar results were observed in a previous study, which showed that the antibacterial activity of AMP B22a against *Vibrio cholera* was accompanied by ROS generation (Rowe-Magnus et al., 2019). However, the exact mechanism needs further investigation.

Drug resistance develops almost at any time antibiotics are routinely used, but few effective approaches seem adequate to overcome this extremely consequential problem. This seemingly helpless situation leaves no choice but to explore new potential alternatives or substitutes for antibiotics. Less drug resistance is the prominent merit of AMPs compared to antibiotics, and thus it is necessary to determine whether novel AMPs from animals or other organisms have the potential to become substitutes for antibiotics. The present study indicated that Spgillcin_177–189_ did not induce any significant resistance in MRSA and MDR *P. aeruginosa* after a long-term (50 days) continuous culture in the presence of sub-inhibitory concentrations of Spgillcin_177–189_. The reasons might be attributed to the rapid action and membrane-based mechanism of the AMP as described by Lazzaro et al. (2020), which makes it difficult for both MRSA and MDR *P. aeruginosa* to acquire drug resistance to Spgillcin_177–189_. Some AMPs have displayed similar results—for instance, the LL-37-inspired peptide SAAP-148 maintained its MIC values of 1.875 μM from the first until the 20th passage (de Breij et al., 2018). In addition, a short peptide derived from fish hepcidin, As-hecp3, had a MIC of 8 μM at the first passage and maintained an almost unchanged antibacterial activity, with MIC at 12 μM after 150 days of continuous culturing of *P. aeruginosa*, confirming that *P. aeruginosa* does not generate significant resistance to As-hec3 (Zhu et al., 2021). In contrast, a MRSA treated with the antibiotic rifampicin resulted in a significant increase in the MIC value of rifampicin from 0.03 μg ml^-1^ at the beginning to 1,024 μg ml^-1^ after 50 days, a result which is consistent with a previous study of *S. aureus* JAR060131 resistance to rifampicin showing a >4,096-fold change in MIC after 20 passages (de Breij et al., 2018). Similar to MRSA, MDR *P. aeruginosa* generated significant resistance to the control antibiotic ceftazidime, with MICs ranging from 2 μg ml^-1^ at the beginning to 128 μg ml^-1^ after 50 days of treatment with ceftazidime. As reported, *P. aeruginosa* usually adopts multiple methods to acquire antibiotic resistance, including modifications of antibiotic targets, formation of biofilms, and overexpression of efflux pump systems (Wyres and Holt, 2018)—for example, the MexCD-OprJ efflux pump system is involved in *P. aeruginosa* resistance to ceftazidime (Fernandez and Hancock, 2012; Sy et al., 2017). A similar severe situation of ceftazidime resistance has now widely occurred in multiple Gram-negative bacteria that produce resistance during the application of third-generation cephalosporin drugs (Tacconelli et al., 2018). Conversely, in this study, MRSA did not produce any significant resistance to the control antibiotic vancomycin (with only a fourfold change in the MIC value) and the AMP LL-37 (with only a twofold change in MIC value). These results also provide support for vancomycin as an excellent drug when employed to kill *S. aureus* and MRSA (Hamamoto et al., 2021), and the short peptide LL-37 also shows admirable antimicrobial effects on some drug-resistant bacteria (Vandamme et al., 2012; Woodburn et al., 2019). Similarly, in this study, MDR *P. aeruginosa* did not show any significant resistance to the control antibiotic polymyxin B (with only a twofold change in MIC value), and this result corresponds to a previous study showing the MIC value of polymyxin B against *P. aeruginosa* ATCC 27853 (Gou et al., 2021). In short, Spgillcin_177–189_ may be used as an alternative for the last-resort antibiotic polymyxin B as a potential antimicrobial agent against MDR *P. aeruginosa* infection.

Biofilms are thought to be an important cause of chronic and recurrent infections but are easily overlooked in antibiotic therapies. Spgillcin_177–189_ shows an anti-biofilm activity against *S. aureus* and *P. aeruginosa* by preventing biofilm formation. This is similar to a previous study of the AMP HPA3NT3-A2, which effectively inhibits the biofilm formation of MDR *P. aeruginosa* (Lee et al., 2019). In accordance with the earlier reports that the minimum biofilm inhibitory concentration is normally lower than the MIC (de la Fuente-Nunez et al., 2016; Raheem and Straus, 2019), Spgillcin_177–189_ exerted anti-biofilm activity at a lower inhibition concentration against either *S. aureus* or *P. aeruginosa* than for bactericidal activity, indicating a different inhibition mechanism on biofilm formation *vs*. killing bacteria. Some serious clinical diseases are caused by *S. aureus* (Lowy, 1998; Klevens et al., 2007)—for example, *S. aureus* is the primary pathogen that survives and persists in macrophages causing a chronic infection (Huo et al., 2020; Pidwill et al., 2020). At present, it is difficult to kill *S. aureus* using traditional antibiotics because this bacterium has developed antimicrobial resistance (Lam et al., 2019). In this study, Spgillcin_177–189_ showed potent antimicrobial effects against *S. aureus* without resistance, and Spgillcin_177–189_ could effectively kill *S. aureus* in the culture supernatant of RAW 264.7 cells. This result was similar to the AMP Ba49, which exhibits a potential bactericidal activity against *S. aureus* in macrophage cells (Taggar et al., 2021), and the AMP CATH-2, which kills *S. aureus* in macrophage cells (Schneider et al., 2017). Therefore, Spgillcin_177–189_ could be exploited as an effective anti-infective drug in the future.

## Data availability statement

The datasets presented in this study can be found in online repositories. The names of the repository/repositories and accession number(s) can be found in the article/**Supplementary Material**.

## Ethics statement

The animal study was reviewed and approved by the Laboratory Animal Management and Ethics Committee of Xiamen University.

## Author contributions

K-JW conceived and designed the experiments and supervised and revised the manuscript. XW and XH performed all the experiments and wrote the original manuscript. K-JW also contributed to all the reagents and materials and wrote the manuscript. FC assisted in the design of the experiments and revised the paper. All authors contributed to the article and approved the submitted version.

## Funding

This study was supported by the National Natural Science Foundation of China (U1805233), the National Natural Science Foundation of Fujian Province, China (grant #2021J05008), the Xiamen Ocean and Fishery Development Special Fund Project (grant #20CZP011HJ06) from the Xiamen Municipal Bureau of Ocean Development, and a grant (grant #3502Z20203012) from the Xiamen Science and Technology Planning Project and Marine Biotechnology Economic Integration Service Platform from Fujian Association for Science and Technology.

## Acknowledgments

We thank the laboratory engineers, Hui Peng and Huiyun Chen, for providing technical assistance. We thank the Second Affiliated Hospital of Fujian Medical University (Quanzhou, Fujian, China) for providing the clinical isolates.

## Conflict of interest

The authors declare that the research was conducted in the absence of any commercial or financial relationships that could be construed as a potential conflict of interest.

## Publisher’s note

All claims expressed in this article are solely those of the authors and do not necessarily represent those of their affiliated organizations, or those of the publisher, the editors and the reviewers. Any product that may be evaluated in this article, or claim that may be made by its manufacturer, is not guaranteed or endorsed by the publisher.
