# Supplementary material for: A truncated peptide Spgillcin177–189 derived from mud crab Scylla paramamosain exerting multiple antibacterial activities
Source: Front Cell Infect Microbiol. 2022 Aug 2;12:928220. doi: 10.3389/fcimb.2022.928220 (PMC9435603; doi:10.3389/fcimb.2022.928220)
Supplement: Supplementary file 1 [file DataSheet_1.pdf]

Supplementary Material

Supplementary Table 1 Specific primers were designed to amplify the *Spgillcin* and detected its expression profiles.

Primer name	Primer sequence (5'-3')
F-*Spgillcin*	ATGTTAGCAAGGGGGGGATCTCCTA
R-*Spgillcin*	TTAGTTGTAGTATGCAGTGAGTGAT
5'*Spgillcin*-R1	GGTGGAAGGGCGACCGTGGCAG
5'*Spgillcin*-R2	TCGAGCCTCGGCGAACGGGC
3'*Spgillcin*-F1	TGGAAGACGAGCGCGCAGGC
3'*Spgillcin*-F2	GGGTGCCTGAGCAGCCCGTC
qPCR-*Spgillcin*-F	ACTGCTGCGAGGTCAACTAC
qPCR-*Spgillcin*-R	GGCGAGAACCAAAGTCTGGA

The expression profiles of Spgillcin

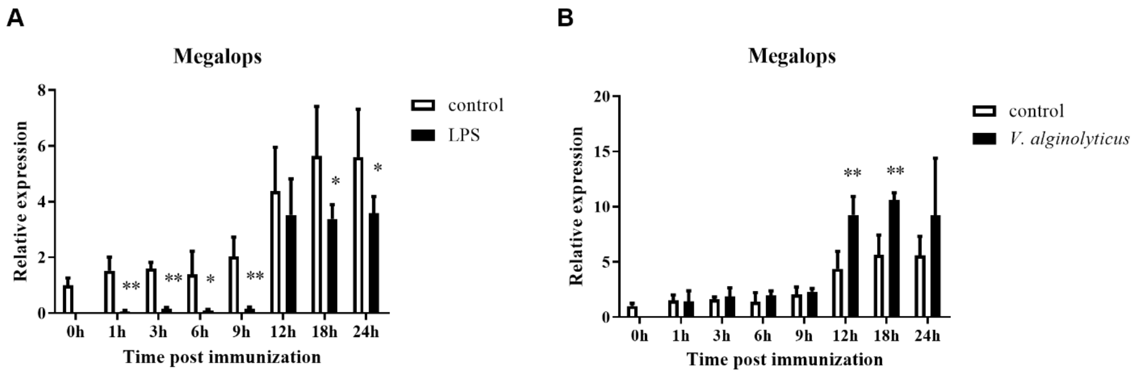

Supplementary Figure 1 The relative expression of Spgillcin was challenged by LPS **(A)** and *V. alginolyticus* **(B)** at megalops stage. Data were represented as mean ± standard deviation (SD). **P* < 0.05 and ***P* < 0.01.

Spgillcin$_{177-189}$ kills *S. aureus* CGMCC 1.2465 and *P. aeruginosa* CGMCC 1.2421 without resistance selection

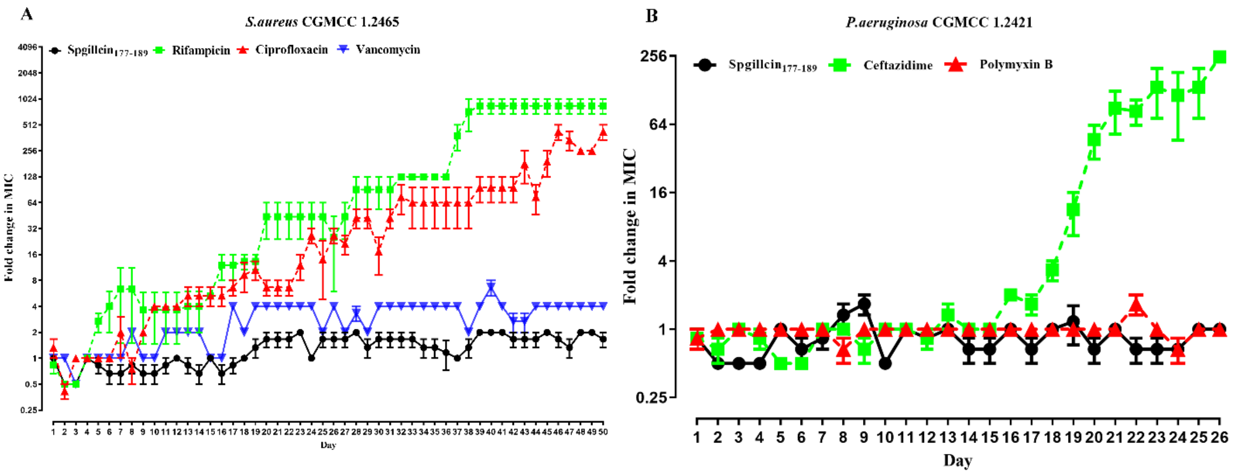

Supplementary Figure 2 Sphillcin$_{177-189}$ killed *S. aureus* CGMCC 1.2465 and *P. aeruginosa* CGMCC 1.2421 without resistance selection. **(A)** Resistance development of *S. aureus* CGMCC 1.2465 after continuous Spgillcin$_{177-189}$, rifampicin, ciprofloxacin and vancomycin treatment. **(B)** Resistance development of *P. aeruginosa* CGMCC 1.2421 after continuous Spgillcin$_{177-189}$, ceftazidime and polymyxin B treatment.
